# Non-Destructive Testing Technology for Shallow Subsurface Defects in Rails: A Review with Focus on Ultrasonic Surface Wave Methods

**DOI:** 10.3390/s26144614

**Published:** 2026-07-21

**Authors:** Tianyu Song, Lisha Peng, Songling Huang, Zijing Huang, Qibo Feng, Hongyu Sun

**Affiliations:** 1 School of Physical Science and Engineering, Beijing Jiaotong University, Beijing 100044, China; 15271391261@163.com (T.S.); qbfeng@bjtu.edu.cn (Q.F.); 2 State Key Laboratory of Power System Operation and Control, Tsinghua University, Beijing 100084, China; penglisha@mail.tsinghua.edu.cn (L.P.); huangsling@tsinghua.edu.cn (S.H.); hzj@mail.tsinghua.edu.cn (Z.H.)

**Keywords:** rail transit, rail, shallow subsurface defects, ultrasonic non-destructive testing

## Abstract

With increasing rail traffic intensity, reliable detection of shallow subsurface rail damage is essential for operational safety. This critical narrative review evaluates non-destructive testing technologies relevant to defects whose active crack front or principal scattering zone lies within the upper approximately 0.5–10 mm of the rail, while treating the 10–15 mm range as a transition to deeper-defect verification. Magnetic flux leakage, magnetic particle inspection, visual inspection, eddy current testing, and conventional ultrasonic testing are first examined as screening or confirmatory comparators. The review then focuses on four ultrasonic surface-wave excitation routes—contact piezoelectric, active air-coupled, electromagnetic acoustic, and laser ultrasonic—and distinguishes source-specific laboratory capability from demonstrated field evidence. Because the cited studies use different defect geometries, rail conditions, sensor configurations, speeds, and decision criteria, their numerical values are reported as source-conditioned evidence rather than as a normalized ranking. An engineering decision matrix links defect depth and size, inspection speed, surface condition, and noise environment to a recommended screening–confirmation workflow. The synthesis identifies contact piezoelectric UT/PAUT as the most mature quantitative confirmation route, while EMAT, air-coupled UT, and laser UT retain method-specific advantages but require stronger natural-defect and in-service validation.

## 1. Introduction

**Figure 1:**
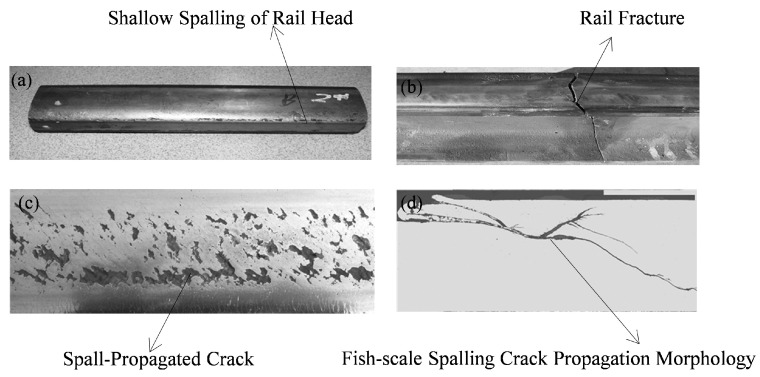
Rail defects: (**a**) shelling crack and shallow spalling; (**b**) transverse fissure and fracture characteristics; (**c**) running-surface shelling crack; and (**d**) fish-scale shelling-crack propagation.

China’s high-speed railway network has expanded rapidly, placing sustained demands on rail inspection. Rails endure repeated wheel–rail contact stresses, friction, and environmental exposure, producing internal, surface-visible, and shallow subsurface damage that can reduce service life and threaten safety [1]. Shallow subsurface defects are especially challenging because the active crack front lies below the running surface and may propagate under cyclic loading before a clear surface indication develops. Reported crack-growth behavior depends on loading, material, and defect geometry, so a single universal critical depth should not be inferred from an isolated study [2]. Although rail-defect taxonomies differ among countries, comparable internal, surface, and subsurface categories recur [3]. In this review, surface wear and profile phenomena—including vertical wear, side wear, corrugation, abrasion, spalling, and indentation—are treated only as visible comparators or triggers for confirmatory inspection; they are not reclassified as buried shallow subsurface defects [4]. The primary target is the shallow subsurface crack front, assessed through a screening–confirmation workflow using magnetic, electromagnetic, visual, and ultrasonic methods. Representative defects are shown in Figure 1.

Scope and terminology are used consistently throughout this review. A surface-visible anomaly is a feature that can be observed directly at the running surface. A surface-connected crack intersects the running surface but may propagate beneath it. The primary term, shallow subsurface defect, denotes a defect whose active crack front or dominant scattering zone lies below the surface within a nominal 0.5–10 mm depth band; the exact interrogated depth remains wavelength- and mode-dependent. Damage extending from 10 to 15 mm is discussed only as a transition band that usually requires bulk-wave UT or PAUT confirmation. Visual inspection and MPI are therefore included as screening comparators, not as direct methods for fully buried shallow subsurface defects.

**Figure 2:**
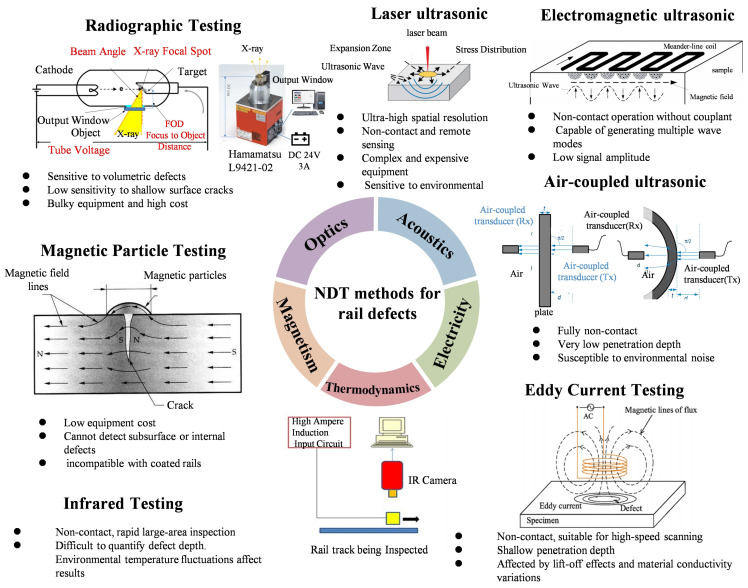
Framework of rail NDT methods classified by physical principle.

Currently, detection methods for various shallow subsurface rail defects include eddy current testing, magnetic flux leakage, magnetic particle inspection, and ultrasonic testing, among others. While magnetic particle inspection offers intuitive defect display and high sensitivity at a low cost, its limitations lie in the requirement for visual inspection by skilled personnel. The method is time-consuming, less automated, and requires expensive, bulky equipment [5]. ECT offers high sensitivity for surface and near-surface defect detection but suffers from lift-off effects [6]. ECT not only places high demands on the equipment but is also limited by the geometry of the test objects. MFL detection can quantitatively analyze the signals from detected defects. Besides, it is vulnerable to nonlinear signal interference arising from the superposition of leakage fields from adjacent defects and environmental noise. These factors jointly limit the accuracy of MFL defect classification [7]. In contrast, ultrasonic testing enables rapid detection and is currently mainly used for internal defects like transverse cracks in the rail head, longitudinal horizontal cracks in the rail head/web, bolt hole cracks, fatigue damage in welded rail heads/webs, and transverse cracks in the rail base projection area of the web. The framework for rail NDT based on different physics branches is shown in Figure 2.

**Figure A1:**
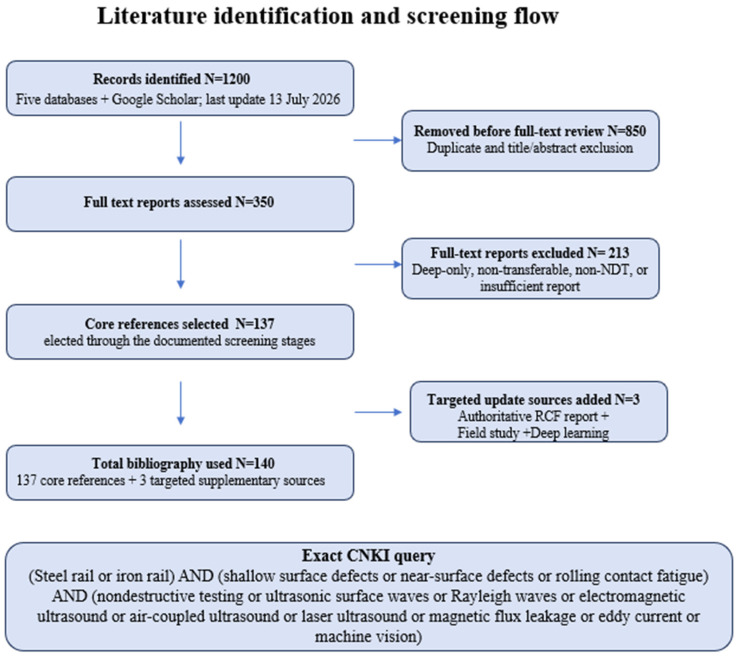
Literature identification and screening flow for the structured narrative review. The 137 core references and three targeted supplementary sources yield the 140-item bibliography; no database-specific duplicate subtotal is invented.

This article is a structured critical narrative review supported by a documented literature search; it is not presented as a prospectively registered PRISMA systematic review. Searches covered publications from 1 January 2000 to 13 July 2026 and were last updated on 13 July 2026 in Web of Science Core Collection, Scopus, IEEE Xplore, ScienceDirect, Google Scholar, and CNKI. Exact database field syntax, date limits, and the evidence-selection flow are provided in Appendix A and Figure A1. Two authors independently screened titles/abstracts and full texts, and disagreements were resolved by discussion.

Eligible evidence comprised peer-reviewed studies directly addressing rail inspection or transferable transducer, wave-propagation, signal-processing, and field-validation methods; standards and authoritative technical reports were retained only as contextual sources. Surface-only studies were included only when they provided a comparator or a transferable method and are identified as such. Duplicate records, studies confined to unrelated deep internal defects, medical/biomedical ultrasound, non-rail work without a defensible transfer pathway, and conference abstracts without an accessible full report were excluded. The retained stage totals were 1200 identified records, 350 full-text reports assessed, and 137 core references selected through the documented screening stages. Thus, 850 records were removed through duplicate removal plus title/abstract screening and 213 reports were excluded after full-text assessment. Three targeted supplementary sources—an authoritative RCF report [8], a full-scale field study [9] and a deep learning reference [10]—were added during the final update, giving a bibliography of 140 sources. Because the original search record did not retain a defensible database-specific duplicate subtotal, the combined value is reported transparently rather than reconstructed with false precision (Figure A1).

## 2. NDT Technologies for Rail Shallow Subsurface Defects

### 2.1. Magnetic Flux Leakage and Magnetic Particle Inspection

**Figure 3:**
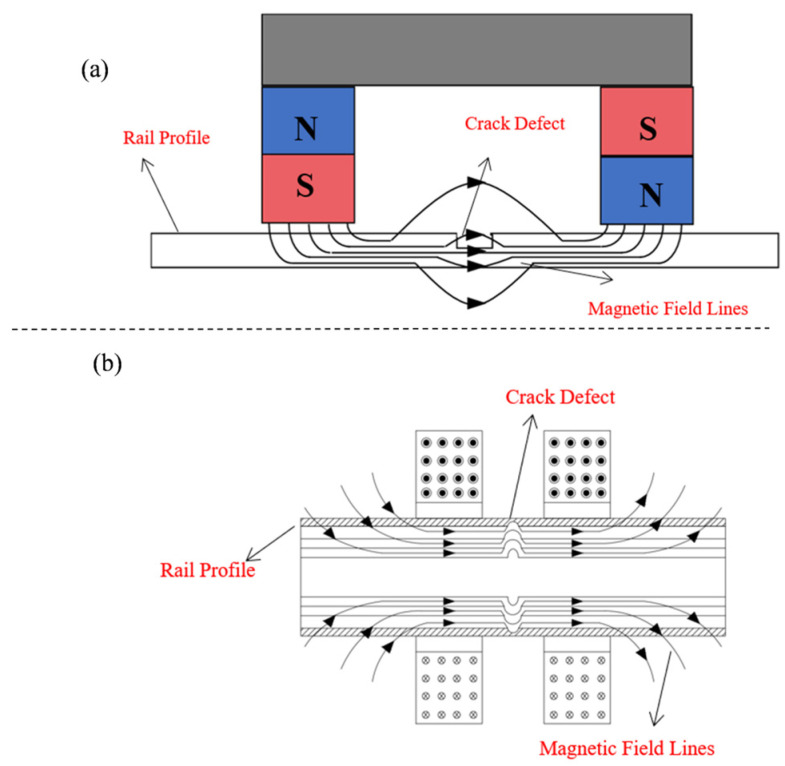
Schematic of the magnetic flux leakage testing principle: (**a**) yoke-type magnetizer; (**b**) encircling coil-type magnetizer.

This section introduces MFL and Magnetic particle inspection (MPI) testing methods. As shown in Figure 3, MFL detection involves magnetizing a ferromagnetic material. Defects alter the flow path of magnetic flux lines within the material, creating leakage magnetic fields at the defect locations. Information about these leakage fields is acquired using magnetic sensors, such as Hall sensors, which enable the detection of surface and subsurface defects in the workpiece. In MFL testing, magnetization methods can be classified primarily by the type of excitation source used: direct current (DC), alternating current (AC), permanent, or hybrid. In recent years, researchers have conducted extensive studies in DC magnetic flux leakage testing, AC magnetic flux leakage testing, and pulsed magnetic flux leakage testing for high-speed rail inspection [11,12,13]. In MFL testing, the inspection signal is influenced by numerous factors. As summarized in Table 1, these parameters include, but are not limited to, defect size, defect orientation, lift-off distance, magnetization strength, applied stress, and scanning velocity. Lift-off, defined as the vertical distance between the magnetic sensor and the surface of the test piece, is a critical parameter. An increase in lift-off during scanning causes rapid attenuation of the measured MFL signal [14].

**Figure 4:**
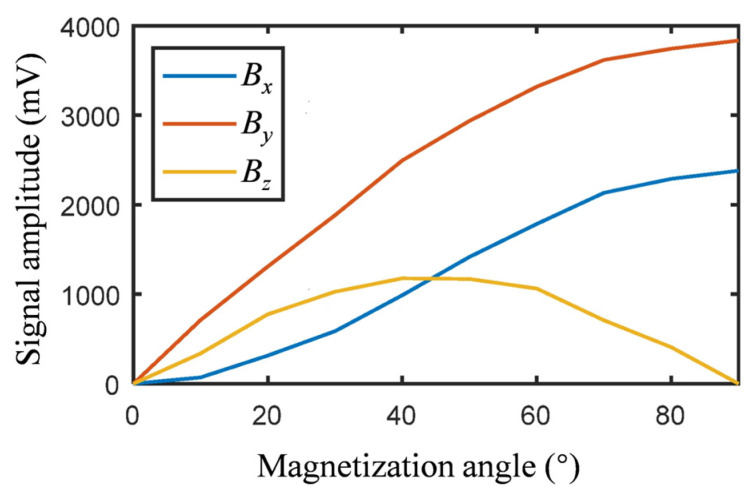
Effect of the angle between magnetization and defect orientation on the MFL response. Reproduced with permission from Wu et al., IEEE Transactions on Magnetics; published by IEEE, 2015 [16].

Within a specific range, the leakage magnetic field intensity is approximately proportional to the defect depth. The influence of defect width is non-monotonic; while the signal amplitude generally increases with crack width, an extensive defect can conversely lead to signal attenuation. It is widely acknowledged that detection sensitivity is highest when the defect orientation is perpendicular to the magnetization field; defects aligned parallel to the field generate negligible measurable leakage fields [15]. Figure 4 illustrates the effect of the angle between the magnetization direction and the defect on the MFL signal, where Bx, By, and Bz are the x, y, and z components of the magnetic flux leakage field, respectively [16].

Strong magnetization is typically required to drive ferromagnetic materials into saturation, which is essential for obtaining a well-defined MFL signal. However, the signal amplitude does not always increase monotonically with magnetizing current. Once the magnetization intensity surpasses a critical threshold, the signal may begin to weaken [17]. The influence of scanning velocity on MFL signals has also been extensively studied, affecting both the signal baseline and amplitude, causing waveform distortion, and generally leading to a decrease in signal amplitude with increasing speed [18,19]. Relative motion between the inspection device and the rail generates two types of motion-induced eddy currents. These eddy currents reduce the peak amplitude of the leakage magnetic field signal and disturb its spatial distribution, particularly affecting the baseline magnetic flux along the transverse and vertical directions of the rail [18]. The inspection equipment may be subjected to vibrations from the surrounding environment during operation, which further interfere with the magnetic leakage signals and degrade detection accuracy [20]. Furthermore, due to magneto-mechanical coupling, the magnetic properties of ferromagnetic materials can be altered under applied stress, consequently modifying the MFL response. In micro-crack detection, surface roughness introduces background noise into the MFL signal, thereby reducing the signal-to-noise ratio (SNR) [21]. Therefore, effectively enhancing the magnetic field generated by surface cracks in rails remains a key challenge for conventional MFL methods.

Early efforts focused on signal enhancement through hardware optimization. Gao et al. [22] developed an MFL detection system based on an 8-bit AT89C51 microcontroller, prioritizing detection accuracy and speed at the system level. To improve sensitivity for minor surface defects, Jia et al. [23]. optimized the sensor structure to reduce vertical vibration interference. However, these hardware-level improvements revealed a critical practical limitation: Although MFL can maintain measurable signal amplitude in laboratory conditions at detection speeds up to 350 km/h, due to noise caused by the speed, crack size characterization significantly deteriorates above 32 km/h [24]. Essentially, MFL signals are easily affected by train speed, underscoring the need for algorithmic interference suppression. This needs motivated lift-off compensation algorithms. Xu et al. [25] proposed a correlation-based filtering algorithm to address the long-standing challenge of MFL signal disturbance caused by lift-off variation. Validated on rail surface defects with aperture sizes from 4 to 7 mm, the algorithm effectively suppressed lift-off interference and achieved an SNR gain exceeding 1.7, demonstrating that algorithmic approaches can compensate for what hardware alone cannot resolve. Current frontier focuses on multi-directional imaging for comprehensive defect characterization. Wilson et al. [26] introduced triaxial magnetic field sensors to enhance defect characterization beyond single-axis measurement. Building on this multi-axis concept, Li et al. [27] proposed a 3D crack profile reconstruction method based on multi-directional magnetic field excitation, fusing MFL information from different excitation directions to achieve more accurate defect geometry recovery. The Key Laboratory of “Nondestructive Testing and Monitoring Technology for High-Speed Transport Facilities” at Nanjing University of Aeronautics and Astronautics further validated MFL detection for various damage types under high-speed conditions using a laboratory turntable simulating 350 km/h inspection speeds, bridging the gap between multi-directional imaging and real-world deployment requirements.

Magnetic particle inspection (MPI) detects discontinuities by magnetizing a ferromagnetic component and applying magnetic particles as a display medium. A discontinuity with a permeability contrast diverts magnetic flux, creating a leakage field and local poles at the surface; accumulated particles then form a visible indication whose geometry reflects the surface expression of the defect. MPI is highly sensitive to surface-breaking cracks and may respond to very shallow buried discontinuities—commonly within about 3 mm under favorable conditions—but sensitivity decreases rapidly with burial depth. It is therefore used mainly for local confirmation of surface-connected or very shallow indications. The minimum detectable crack width depends on particle size, magnetization direction and strength, surface condition, and observation conditions [28].

As shown in Table 2, factors affecting MPI sensitivity and image quality include the geometry and surface condition of the workpiece, the magnetic properties of the material, the magnetization technique, and the characteristics of the magnetic particles used. The distribution of the magnetic leakage field influences the movement of magnetic particles, thereby affecting their accumulation. When the magnetizing field strength is sufficiently high, a strong enough leakage field is generated on the workpiece surface to attract the magnetic particles [29]. However, a higher magnetization intensity is not always better; it must be optimized based on the workpiece material and dimensions, as excessively high magnetization may lead to excessive background particle adherence. Magnetization methods include circumferential, longitudinal, and multi-directional magnetization. Selecting the appropriate magnetization method is a prerequisite for effective defect detection. Due to the skin effect, AC offers high sensitivity for surface defects, while DC or half-wave rectified DC provides greater penetration depth. However, numerous properties of the magnetic particles, including their size, concentration, shape, magnetic properties, mobility, visibility, and durability, significantly influence their effectiveness [30]. If the particle concentration in the carrier fluid is too low, insufficient particles accumulate at defect sites, leading to faint indications and potential missed detections. Conversely, if the concentration is too high, many particles may adhere to the workpiece surface, creating a high background that can mask defect indications [30]. The particle size distribution and shape also affect their accumulation at defect locations, thereby influencing the visual characteristics of the magnetic particle indications. Only ferromagnetic materials are suitable for magnetic particle inspection. Furthermore, the smoothness and cleanliness of the workpiece surface affect particle movement and accumulation. Surface-breaking defects with greater depth are more likely to produce significant leakage fields. Generally, the magnetic leakage field signal intensifies with increasing defect depth, while it gradually weakens with increasing distance from the defect [30].

Despite advancements in equipment integration technology, MPI remains highly reliant on the operator’s expertise. With the development of computer vision, deep learning methods enable automatic defect detection, localization, and classification by analyzing digital images, allowing for the identification of subtle defects even against complex backgrounds [31,32]. These advances highlight the feasibility and potential of automating MPI through image processing and computer vision techniques. Nevertheless, developing robust algorithms that adapt to variations in surface conditions and complex specimen geometries remains an ongoing challenge in MPI [33]. Chen et al. proposed a three-dimensional profile-measurement-based magnetic particle testing (3DPMPT) method for automatic crack identification [34]. This approach detects cracks by analyzing the profile variations induced by magnetic particles on the tested object, thereby overcoming the limitation of traditional MPT, which relies on enhancing the visual characteristics of magnetic particles. Consequently, 3DPMPT can avoid constraints associated with conventional MPT, such as stringent lighting conditions, environmental contamination, and the need to treat magnetic particles chemically.

### 2.2. Visual Inspection

Visual inspection directly senses surface-visible rail anomalies; it cannot by itself detect a fully buried shallow subsurface defect. It is retained in this review as a high-speed screening comparator because surface manifestations—such as crack mouths, spalling, shelling, corrugation, and wear—can trigger ultrasonic confirmation. Industrial cameras, laser scanners, or multispectral cameras acquire the running-surface condition, often with encoder-synchronized illumination to reduce motion blur. Image preprocessing, texture and gradient analysis, structured-light or laser-triangulation profilometry, stereo reconstruction, and machine-learning classification can quantify visible crack patterns and surface topography. The resulting depth estimate describes surface relief rather than the buried crack-front depth; this distinction is essential when visual results are compared with ultrasonic shallow-subsurface measurements.

**Figure 5:**
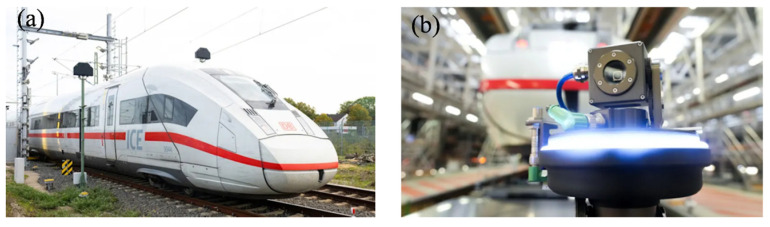
E-Check system for ICE train inspection: (**a**) ICE high-speed train; and (**b**) ACE 2 camera in Deutsche Bahn’s E-Check system.

Famous rail inspection vehicles, such as ENSCO VIS Integrated Rail Inspection System and Germany’s Alias Elektronik Rail Check (Figure 5), utilize advanced camera technology to capture images for automatic fault identification in railway systems [35]. Li et al. proposed a double-layer data-driven framework for automatic visual inspection of rail surface cracks [36]. The effectiveness of this framework was validated using real rail images provided by China Railway Corporation and the Hong Kong Mass Transit Railway (MTR). Comparative experiments with six benchmark methods—including Otsu’s method, mean shift, visual detection systems, geometric approaches, fully convolutional networks, and U-Net—demonstrated that the proposed framework achieves the best performance in rail surface crack detection. Yan et al. proposed generating a latent normal image by learning the characteristics of normal rails using an improved Pix2Pix network [37]. Defect regions were inferred by comparing the input detection image with the generated latent normal image. The experimental results showed that mean pixel accuracy (MPA) reached 0.9984 and mean intersection over union (MIOU) reached 0.8305. Xu et al. proposed a lightweight algorithm based on improved YOLOv8, redesigning the neck architecture by integrating feature extraction, fusion, and injection modules [38]. They replaced the traditional Complete Intersection over Union (CIOU) with Scylla-IOU (SIOU) to enhance detection performance. The algorithm achieved 94.9% precision and 81.9% recall, with a 26.3% increase in frames per second (FPS) and a reduction in model size to 63% of the original.

Standalone visual inspection is now rarely used on its own and is typically used in conjunction with other methods. With the increasing integration of artificial intelligence (AI), particularly machine learning (ML) and deep learning (DL), visual inspection systems are becoming more intelligent. These systems can automatically extract features by learning from large volumes of sample images, thereby significantly enhancing their ability to recognize complex, variable defects.

### 2.3. Eddy Current Testing

**Figure 6:**
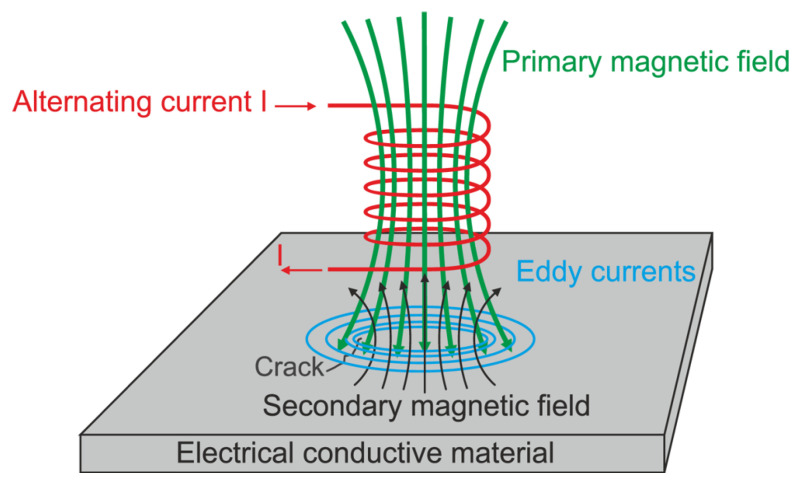
Schematic of eddy-current testing. Reproduced with permission from Pohl et al., NDT & E International; published by Elsevier, 2004 [54].

ECT is based on the law of electromagnetic induction. An energized excitation coil induces eddy currents in a conductive workpiece. As shown in Figure 6, defects within the workpiece alter the eddy-current field intensity, thereby changing the impedance and other parameters of the detection coil. These changes provide information about defect characteristics. ECT devices typically consist of excitation and detection coils. Researchers have improved detection devices by modifying sensor structures and coil shapes. ECT technology has advantages like being mature, non-contact, high-speed, and adaptable to harsh environments, making it an ideal choice for real-time detection. It supports fast scanning, and the data generated is inherently quantitative, requiring very little computation for later analysis [39]. These advantages make ECT a valuable, highly adaptable nondestructive testing technique suitable for a wide range of industries and applications.

As summarized in Table 3, excitation frequency, lift-off, electrical conductivity, magnetic permeability, temperature, and component thickness all affect eddy-current testing (ECT) performance [40]. Excitation frequency controls the electromagnetic penetration depth: lower frequency increases penetration but generally reduces sensitivity to small surface discontinuities, whereas higher frequency improves near-surface sensitivity [41,42]. Lift-off changes the probe impedance as the coil–surface distance varies and can mask a crack response when its signal trajectory overlaps the defect indication [43,44,45]. Probe shape and frequency must therefore be matched to component geometry and thickness [44]. Material conductivity and permeability govern the magnitude and distribution of induced currents, while local changes caused by a discontinuity provide the detection contrast [46,47]. Workpiece shape, thickness, and diameter can introduce additional background signals. Crack orientation is also important because ECT is most sensitive when the defect interrupts the eddy-current flow [48]. Motion-induced eddy currents and electromagnetic drag become more pronounced with speed and depend inversely on conductivity and permeability, which can shift the signal baseline and reduce usable sensitivity under dynamic conditions [49,50].

To improve rail-defect classification, Wang et al. evaluated an eddy-current inspection system using method-specific artificial rail specimens [51]; the specimen geometries and laboratory conditions do not define a universal minimum detectable size. Alvarenga et al. developed an embedded system that applies convolutional neural networks to wavelet-transformed eddy-current signals for online localization and classification [52]. In that study, three anomaly categories—squats, welds, and joints—were classified with a reported accuracy of 98%. Mussatayev et al. proposed a hybrid probe combining a transmit coil with a differential receive coil to suppress lift-off noise. In controlled tests, a figure-eight driver configuration produced a reported SNR above 100 for an artificial defect measuring 1 mm × 1 mm [53]. These values remain conditional on the cited probe geometry, lift-off, surface condition, and decision threshold.

### 2.4. Ultrasonic Testing (UT)

Ultrasound is a mechanical vibration wave above 20 kHz. Its directivity and penetration in solids make ultrasonic testing a mature, radiation-free technique for rail inspection [55]. Ultrasonic guided waves can propagate over extended distances while remaining sensitive to discontinuities near the rail surface. Rayleigh-wave displacement is concentrated within a wavelength-dependent zone beneath the free surface, which makes the mode suitable for surface-connected cracks and shallow subsurface defects when the frequency is selected for the target depth. Lamb waves are mainly associated with plate-like structures, where symmetric and antisymmetric modes are selected through the frequency–thickness product.

Guided wave testing is an NDT technique that utilizes guided waves propagating under structural constraints for long-distance, large-area inspection. Its core principle is based on the interaction between the propagation characteristics of guided waves in specific geometries and defects. Guided waves are elastic waves propagating in finite media, constrained by the structure’s geometry, with energy propagating along the structure’s length rather than radiating outward. Because different wave modes propagate at characteristic speeds, when ultrasound propagates within a bounded medium such as a rail, various reflected, transmitted, refracted, and interface waves couple, forming ultrasonic guided waves. Dang et al. proposed a novel Guided Wave Testing (GWT) method using optical fiber sensing combined with Orthogonal Matching Pursuit (OMP) based data processing. It detects rail damage by reconstructing defect-related reflected waves from complex raw signals [56].

UT has two detection methods. One is contact detection, which typically uses a contact transducer to receive the ultrasound signal. This method directly contacts the measured object, providing good acoustic coupling. The detection data is stable with minimal noise interference, resulting in high reliability [57]. However, using contact transducers requires couplers, which limit detection coverage and make them unsuitable for detecting certain objects [58]. The other method is non-contact detection, such as laser ultrasound testing (LUT).

As shown in Table 4, the parameters influencing ultrasonic testing are analyzed from four principal aspects: acoustic properties, equipment parameters, defect characteristics, and inspection conditions. Inspection sensitivity refers to an ultrasonic testing system’s ability to detect the smallest flaws within a material, and its configuration requires careful consideration of factors such as the instrument, probe, material properties, and defect type [59]. The selection of frequency depends on the required sensitivity, ultrasonic penetration depth, or desired resolution [60]. A higher ultrasonic frequency results in a shorter wavelength, which enhances the resolving power for fine defects; however, it also leads to poorer penetration capability and more rapid energy attenuation. Conversely, a lower frequency yields a longer wavelength and greater penetration depth, but at the expense of reduced inspection sensitivity [61,62,63]. The distance of a defect from the ultrasonic probe significantly affects the amplitude of the received signal. The signal amplitude is directly related to the reflection strength from the defect and serves as a critical basis for evaluating defect severity and conducting quantitative analysis. Beams with different angles are used to detect flaws oriented at various angles relative to the inspection surface; for example, straight beams are primarily employed for defects parallel to the inspection surface [61,62]. The choice of probe type depends on the defect orientation and the workpiece geometry. The strongest reflected signal and the highest detection sensitivity are achieved when the ultrasonic wave propagates perpendicular to the defect surface. Larger defects generally produce stronger echo signals, and defects of different natures exhibit distinct acoustic impedance characteristics, leading to distinct echo signal features [60]. The acoustic properties of a material mainly include the propagation velocity of ultrasound within the material and the energy loss during wave transmission. Sound velocity is a key parameter for defect localization and material characterization, while attenuation is related to the material’s microstructure; excessive attenuation reduces the penetration depth and signal-to-noise ratio of the inspection [60]. Complex geometries may generate interfering echoes, complicating the identification of defect signals. The type of couplant, its layer thickness, and the smoothness of the contact surface directly influence the efficiency of acoustic energy transmission. Unstable coupling can cause significant variations in signal amplitude, compromising the reliability of the inspection results. Furthermore, the method’s reliance on couplants, which are susceptible to extreme weather conditions and surface irregularities, presents another significant constraint. Experimentally verified inspection speeds with conventional ultrasonic NDT range only from 40 to 80 km/h, with real-world speeds potentially of 15 km/h, especially during manual verification of the vehicle [64]. Consequently, these drawbacks impede the further development and application of traditional ultrasonic testing techniques.

**Figure 7:**
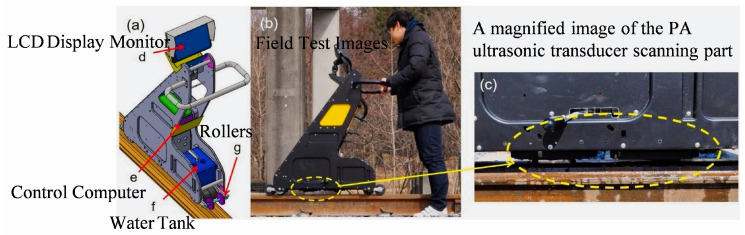
Phased-array ultrasonic transducer: (**a**) its schematic, (**b**) field test images, (**c**) a magnified image of the PA ultrasonic transducer scanning part, (d) LCD display monitor, (e) control computer, (f) water tank, and (g) rollers. Reproduced with permission from Kim et al., Sensors and Actuators A: Physical; published by Elsevier, 2020 [66].

However, conventional ultrasonic techniques, such as the normal-incidence pulse-echo method, struggle to resolve echoes from near-surface defects. This is because the reflections from such defects often fall within the time duration of the transmitted ultrasonic pulse. As a result, the minimum detectable depth of a defect is limited by the probe frequency [65]. Conventional linear array ultrasonic phased array technology controls the time delay of excitation pulses for each element in the transducer array. This changes the phase relationship of the waves emitted by each element when they reach a specific point inside the object, enabling changes to the focus point and beam steering for scanning and imaging, and enabling the detection of sub-millimeter defects. Phased array UT controls multiple elements to independently emit ultrasound of the same frequency. The interference and superposition of these waves in space achieve beam focusing or steering, making it suitable for defect detection in complex geometries. Kim G et al. developed an integrated Phased Array Ultrasonic System for Rails (PAUSR) [66]. They optimized acoustic field parameters using CIVA simulation and experimentally validated the system. Crack detection results (e.g., star cracks in bolt holes, longitudinal vertical cracks) highly matched radiographic testing and simulation results, improving the accuracy and efficiency of internal rail defect detection. The system schematic is shown in Figure 7.

Sun et al. combined nonlinear sideband peak counting with pulse-echo testing for rail fatigue evaluation [67], while Wang et al. developed contact pulse-echo nonlinear imaging using a dual-crystal probe [68]. Sudharsan et al. fused PAUT and pulsed-thermography data in an automatic defect-recognition system (M-ADR) [69]. The TmR-CNN was trained and tested using 125 finite-element datasets (65 side-drilled holes and 60 cracks; 80/20 split). Within that study, the reported overall accuracy and F1-score were 91.46% and 91.62%, respectively; precision/recall were 92.3%/88.2% for side-drilled holes and 93.5%/91.5% for cracks, with reported false-positive rates of 15.0% and 7.7%. Quantitative sizing errors using the Bi-MAT method ranged from 1.65% to 8.65%. Validation on 14 fabricated side-drilled holes and three fabricated cracks provided limited proof-of-concept agreement between simulation-trained and controlled experimental data. The small experimental sample, absence of natural rail defects, possible dependence between simulation cases, and lack of independent in-service validation mean that these results do not establish field generalizability.

### 2.5. Synthesis 

Table 5 does not treat the largest number reported for each technique as a normalized method-level capability. Inspection-platform speed, signal-acquisition speed, and the speed at which defect performance was validated are different quantities. The cited MFL literature reports up to 180 km/h for a summarized screening system [70] and ≤60 km/h for a large inspection vehicle [23]; VT acquisition up to 400 km/h concerns large surface-visible anomalies rather than buried-crack performance [71]; and ECT evidence spans 5–30 km/h for one vehicle study [72]. A statistically valid normalization would require a common rail grade, natural/artificial defect set, sensor geometry, environment, and probability-of-detection and false-alarm criteria. These data are unavailable across the cited studies, so Table 5 is an evidence-traceability table.

## 3. Evolution of Ultrasonic Surface-Wave Detection Technology for Shallow Subsurface Defects

### 3.1. Technical Principle and Suitability for Shallow Subsurface Detection

**Figure 8:**
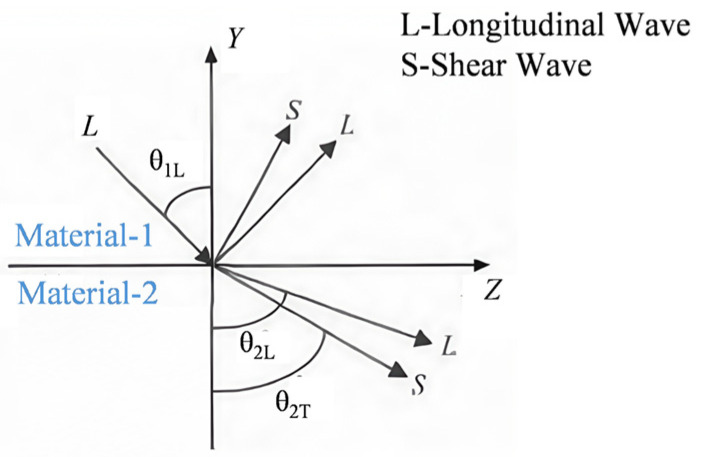
Wave-mode conversion at an interface.

When alternating stress acts at the surface of a medium, Rayleigh waves propagate along the free surface. At an interface, ultrasonic waves undergo reflection, transmission, refraction, and mode conversion according to the material properties and incidence angle, as shown in Figure 8. Rayleigh-wave particle motion is elliptical and combines longitudinal and transverse components. The displacement amplitude decreases rapidly with depth, and the practically interrogated zone is governed by wavelength, mode content, and the required signal-to-noise ratio rather than by a fixed universal depth. Surface waves are therefore well suited to surface-connected cracks and shallow subsurface defects when the excitation frequency is chosen so that the wave field overlaps the target depth.

### 3.2. Direct Excitation by Piezoelectric Transducers

The principle of direct ultrasonic excitation by piezoelectric transducers is based on the electromechanical energy conversion process of the piezoelectric effect. Its core is converting electrical energy directly into mechanical vibration using the piezoelectric effect. Piezoelectric materials possess two key characteristics: the direct piezoelectric effect and the inverse piezoelectric effect. The direct piezoelectric effect generates charge under mechanical stress and is used for receiving ultrasound. The inverse piezoelectric effect causes mechanical deformation in response to an applied electric field and is used to transmit ultrasound. Ultrasound is primarily excited by the inverse piezoelectric effect. A piezoelectric transducer connected to a high-frequency AC power source receives an alternating voltage (e.g., a sine wave or pulse signal), generating a rapidly changing electric field within the piezoelectric material. The electric dipoles within the material undergo directional motion under an electric field, causing periodic expansion/contraction or shear deformation. This deformation is transmitted to the surrounding medium as mechanical vibration. The vibration of the piezoelectric material is transmitted to the test medium through a coupling layer, forming longitudinal or shear waves. When the vibration frequency matches the input electrical signal frequency, an ultrasound of a specific frequency is generated. Compared to other UT methods, PAUT utilizes piezoelectric crystal transducers in a single assembly. Each transducer can be pulsed at different times to achieve constructive interference and individually focused without steering the probe [66]. Due to its powerful scanning capability, it can meet the requirements for full-section detection of 75 kg/m rails, which is beneficial for defect detection in heavy-haul railways [81].

Li et al. proposed a surface crack measurement method based on Rayleigh wave analysis in the frequency domain, calculating crack depth using the reflection factor determined from Rayleigh waves scattered at surface fatigue cracks, successfully monitoring cracks in the depth range of 0.36–0.94 mm [82]. Wong KV et al. proposed an ultrasonic Structural Health Monitoring (SHM) method for assessing defects on the same surface and side as a piezoelectric ultrasonic transducer array, offering an effective approach for monitoring near-surface defects in metals [83]. Kim et al. developed a PAUT system to detect rail cracks. The system can evaluate crack sizes larger than 2 mm [66]. Gao et al. used a fiber-optic picosecond laser as an excitation source for PAUT to detect cracks in steel blocks. The data show that the phased-array laser source significantly improves the amplitude and signal-to-noise ratio of the diffracted signals compared to a single-laser source [84].

**Figure 9:**
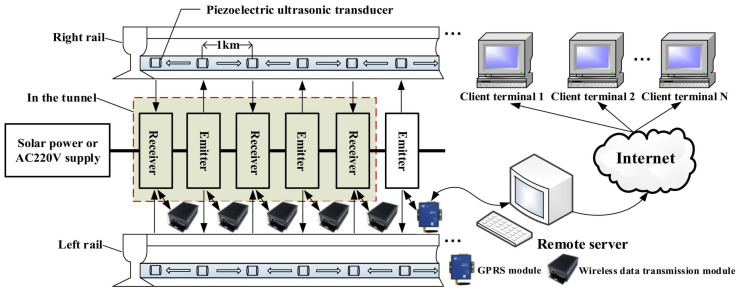
Schematic of a broken-rail detection system based on ultrasonic guided-wave technology. Reproduced with permission from Yang et al., Sensors and Actuators A: Physical; published by Elsevier, 2023 [86].

In the application of rail nondestructive testing, the performance of piezoelectric ultrasonic transducers is influenced by numerous factors. These include selecting piezoelectric materials and designing the transducer structure (e.g., frequency, bandwidth, and the dimensions and shape of the piezoelectric element). The energy transfer across the transducer–workpiece interface is governed by the acoustic impedance mismatch. Because the acoustic impedance of air (Z ≈ 4 × 10^2^ kg·m^−2^·s^−1^) is roughly five orders of magnitude lower than that of steel (Z ≈ 4.6 × 10^7^ kg·m^−2^·s^−1^), even a microscopic air gap reflects over 99.9% of the ultrasonic energy. Coupling media (gels or water) bridge this impedance gap, raising the transmission coefficient to above 50%, which is why piezoelectric transducers achieve the highest energy conversion efficiency among all four methods but require physical contact. The size of the piezoelectric element significantly influences the acoustic beam’s directivity and the length of the near-field region. Larger elements generate a more focused beam with higher energy density, resulting in improved directivity and being advantageous for defect localization. However, the near-field length scales with the square of the piezoelectric wafer diameter, thereby extending the near-field region. Since the effective detection range typically begins at 1 to 1.5 times the near-field length, this characteristic makes larger elements less favorable for detecting shallow subsurface defects within 0.5 to 5 mm beneath the rail surface. Additionally, factors such as the choice of couplant and coupling method, the surface condition of the workpiece being tested, and digital signal processing are all critical considerations in piezoelectric ultrasonic testing. A significant drawback of the standard piezoelectric ultrasonic testing method using a transducer pair is its poor discrimination capability. It is challenging to determine whether a measured signal variation is due to a genuine change in the specimen’s integrity or to extraneous influences. These influences encompass environmental factors, such as temperature changes, and system-related factors, such as adhesive and transducer aging. This fundamental ambiguity, stemming from the lack of a stable reference, poses a major challenge for accurate damage diagnosis and long-term monitoring [85]. Under harsh operating conditions, Ultrasonic Guided Wave (UGW) systems can reliably detect defects in continuously welded rails. A UGW-based broken rail detection system is shown in Figure 9. It consists of a transmitter, receiver, communication equipment, power supply, a Single Piezoelectric Ultrasonic Transducer (SPUT), a remote server, and a client terminal. The system employs sandwich piezoelectric ultrasonic transducers (SPUT) to generate and receive UGW signals traversing the rail in the longitudinal vibration mode. The operating resonance frequency of these SPUTs is 33.69 kHz, and studies have found that UGWs at f = 35.6 kHz are particularly suitable for transmission over long rail networks. This inspection system uses a pitch-capture configuration, with SPUTs functioning both as transmitters and receivers, deployed along continuously welded rails, with a detection distance of approximately 1 km between adjacent transducer pairs. To address issues such as impedance mismatch and frequency shift of the SPUT and low transmission efficiency, the system combines resonance frequency tracking and broadband electrical impedance matching (EIM) at the transmitting end, significantly improving the power transfer efficiency of the SPUT.

### 3.3. Air-Coupled Surface Wave Excitation

**Figure 10:**
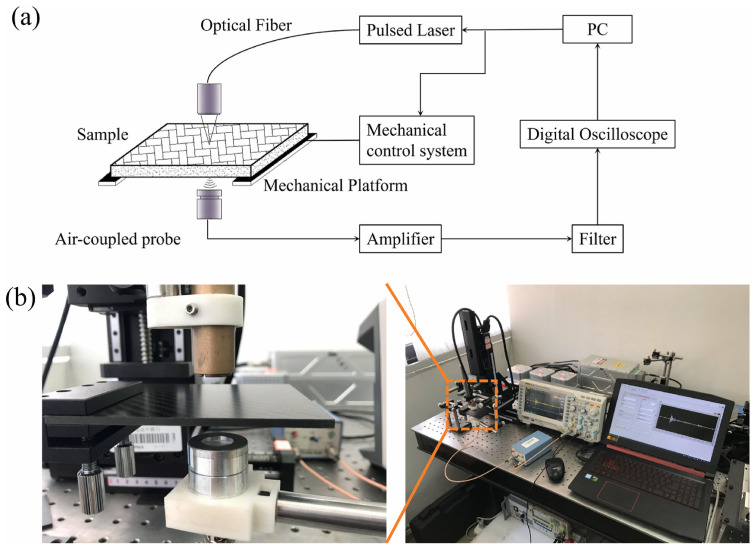
Non-contact laser–air-coupled ultrasonic inspection system: (**a**) schematic and (**b**) experimental setup. Reproduced with permission from Zeng et al., Composites Communications; published by Elsevier, 2021 [88].

Active air-coupled surface-wave excitation is a non-contact ultrasonic technique in which airborne longitudinal waves are incident at an angle selected for mode conversion into Rayleigh or guided waves in the solid. It can interrogate surface-connected or shallow subsurface discontinuities without a liquid couplant. The cited air-coupled transducers commonly operate in the 0.75–2 MHz range [87], although lower frequencies are often required to reduce air-path attenuation in rail-steel applications. The transmitter and receiver may be arranged in pitch–catch or through-transmission configurations; these active configurations must not be conflated with passive air-coupled receivers that exploit wheel–rail excitation. Figure 10 shows a non-contact laser–air-coupled laboratory system used on non-rail composite specimens [88], which is included only as transferable transducer evidence.

Zhou et al. developed a novel air-coupled ultrasonic transducer featuring a plano-concave acoustic lens and dual matching layers [89]. This focused transducer demonstrated 45.0% higher sensitivity than flat-plate transducers in detecting defects in carbon fiber reinforced plastic (CFRP) composites. Bustamante et al. employed a point-focused piezoelectric air-coupled transducer for C-scan testing of artificial defects in aluminum plates, carbon fiber composite boards, and epoxy resin boards [90]. Their system successfully identified hole defects with diameters of 1–2 mm. These studies demonstrate that air-coupled ultrasonic techniques are predominantly applied for defect detection in composite materials.

Owing to substantial disparities in acoustic properties between air and solid materials, this technology faces numerous challenges. The advancement of air-coupled ultrasonic technology necessitates overcoming two pivotal physical constraints: high impedance mismatch and high attenuation [91]. Air exhibits an extremely low acoustic impedance, whereas the acoustic impedance of most solid materials under test, such as metals and composites, is remarkably high. When acoustic waves propagate from air into a solid, upwards of 99.99% of the wave energy is reflected, with only a minuscule portion penetrating the solid. During the propagation of acoustic waves, their energy gradually dissipates. Additionally, acoustic attenuation in air scales with the square of frequency, severely limiting the usable bandwidth. These two factors—interfacial reflection and air-path attenuation—force air-coupled systems to operate at low frequencies (typically <1 MHz) with high-voltage excitation, resulting in inherently low sensitivity and spatial resolution. The greater the distance between the probe and the specimen, the more pronounced the signal loss becomes. This significantly limits the available ultrasonic frequencies and the detection range. Therefore, when air-coupled ultrasonic surface wave detection is performed, the echo signal must be amplified [92]. These problems can be addressed by optimizing the transducer design, refining the electronic system and signal processing algorithms, and making judicious selections of detection parameters.

### 3.4. Electromagnetic Acoustic Transducer (EMAT) Surface-Wave Excitation

**Figure 11:**
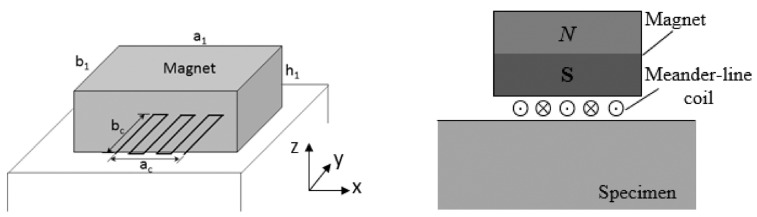
Schematics of electromagnetic acoustic transducer principles. Reproduced with permission from Pei et al., Sensors and Actuators A: Physical; published by Elsevier, 2016 [93].

Typically, an EMAT based on the Lorentz force mechanism includes a coil of wire, a permanent magnet, and a conductive metal. EMAT surface wave excitation operates via the Lorentz force mechanism and the magnetostriction effect (in ferromagnetic materials). It induces mechanical vibrations on the surfaces of conductive or ferromagnetic materials via alternating electromagnetic fields, thereby directly generating surface waves (Figure 11) [93]. Excitable waveforms include Rayleigh waves, Lamb waves, spiral shear horizontal (SH) waves, etc. A planar or meander coil energized by high-frequency alternating current produces an alternating magnetic field, while permanent magnets or electromagnets supply a static magnetic field to enhance magnetostriction. The alternating magnetic field induces eddy currents in the material’s surface layer. Interaction between these eddy currents and the static magnetic field generates Lorentz forces that drive surface particle vibrations. Concurrently, the alternating magnetic field induces periodic lattice expansion/contraction, thereby amplifying the surface wave amplitude. The pulser/receiver is an electronic device that generates high-voltage electrical pulses to drive the transducer to produce high-frequency ultrasonic energy. The ultrasonic frequency for rail flaw detection is 0.2~25 MHz, and the most commonly used is 0.5~10 MHz [94,95]. The same EMAT coil operates in pulse-echo mode (simultaneous transmission and reception).

EMAT is an entirely non-contact technique and does not need any couplants. Moreover, it is less affected by surface conditions. EMATs generate ultrasound through electromagnetic–mechanical conversion: an alternating current in the coil induces eddy currents confined to a thin skin layer, and the static magnetic field converts these currents into Lorentz forces that launch elastic waves. This three-step conversion chain—electromagnetic → mechanical force → elastic wave—fundamentally limits the efficiency to typically 0.1–1%. Furthermore, the induced field decays exponentially with coil-to-sample distance, so that a few millimeters of lift-off can attenuate the signal by over an order of magnitude. The non-contact advantage therefore comes at the cost of low conversion efficiency and strong lift-off sensitivity. Conventionally, EMAT is kept at a 3 mm lift-off to achieve a sufficient SNR. To address challenges in practical electromagnetic ultrasonic testing, such as low energy conversion efficiency, inflexible adjustment of transmission angles, and large transducer size, Sun et al. proposed an SH guided wave EMAT utilizing a double-layer staggered oblique folding (DSOF) coil for detecting cracks in train axles. Compared to traditional coil EMATs, this novel transducer structure more than doubles the intensity of the excited ultrasonic signal, halves the wave packet width, and allows flexible control of the spiral guided wave’s transmission angle by adjusting the excitation current [96]. Wang et al. proposed a miniaturized EMAT optimized for detecting surface defects in thick steel plates. They used an orthogonal experimental design to optimize key design parameters, including magnet geometry, coil configuration, and lift-off distance. This resulted in a 52.4% reduction in volume and a 4.9% increase in signal amplitude. However, this miniaturization inevitably led to performance degradation. To mitigate this effect, they employed a complementary ensemble empirical mode decomposition algorithm [97]. Lan et al. developed a Halbach-array-based permanent-magnet structure, demonstrating enhanced unidirectional surface-wave performance in magnetic-field distribution, eddy currents, Lorentz forces, and displacement fields [98]. Their simulations quantified micro-crack responses with improved defect characterization. Hu et al. investigated scattering characteristics and time-domain echo signals of low-frequency (0.3 MHz) surface waves interacting with inclined tread cracks of varying depths [99]. Their surface-wave EMAT design incorporated a stationary wavelet transform (SWT) for denoising and B-scan reconstruction, improving inspection efficiency while compensating for rail surface unevenness.

In rail nondestructive testing, the performance of EMAT surface-wave testing technology is influenced by multiple factors. The generation and detection of ultrasonic modes depend on the EMAT coil and magnet configurations. The coupling between the electromagnetic and elastic fields is strongly affected by the distance between the EMAT and the material surface. The wavelength of the EMAT coil directly determines the wavelength and frequency of the excited surface waves, while its shape influences the directivity and mode purity of the acoustic beam [100]. Secondly, the magnetic field strength, orientation, and magnetic circuit design of the permanent magnet collectively govern the efficiency of both the Lorentz force and magnetostriction mechanisms. When considering the Lorentz force as the dominant transduction mechanism, the amplitude of the received and generated signals is directly proportional to the strength of the biasing magnetic field [101,102]. Thirdly, the excitation current frequency influences the resolution of the generated surface waves, and the alternating current skin depth at the conductor surface affects the interaction range between the electromagnetic field and the rail, with coupling efficiency highly dependent on the material’s electrical conductivity and magnetic permeability. Additionally, an increase in lift-off results in exponential decay of transduction efficiency and alters the amplitude of the generated signal, thereby changing its magnitude. The generated surface waves are also susceptible to waveform distortion. Variations in impedance due to lift-off result in a phase shift in the output voltage, leading to velocity measurement errors. The magnetostriction generation mechanism exhibits similar behavior [103,104,105].

### 3.5. Laser Ultrasonic Surface-Wave Excitation

Laser ultrasonic surface wave excitation is a non-contact, high-precision ultrasonic surface wave generation technology that utilizes the interaction between a laser and materials to produce surface acoustic waves, with its primary principles based on the thermoelastic and ablation effects. When a pulsed laser irradiates the material surface, it induces localized thermal expansion or plasma eruption, thereby exciting surface waves. Depending on the laser energy density, the process involves two distinct mechanisms. The thermoelastic effect occurs when absorbed laser energy is converted into heat, causing thermal expansion that generates stress waves that propagate and transform into Rayleigh waves. The ablation effect, on the other hand, involves surface vaporization, forming plasma that creates shock waves; these plasma shock waves act on the material surface to generate high-intensity surface waves. In the thermoelastic regime, a laser pulse heats a thin surface layer (thermal penetration depth ≈ 1 μm for a 10 ns pulse in steel), and the resulting rapid thermal expansion generates ultrasound. The process involves four conversion steps—optical absorption → thermal → mechanical → elastic wave—and only 30–40% of the incident optical energy is absorbed (the remainder is reflected by the metallic surface), yielding an overall efficiency of 10^−6^–10^−4^. The method thus offers truly remote, non-contact excitation but is intrinsically low in energy conversion and highly sensitive to surface optical properties.

**Figure 12:**
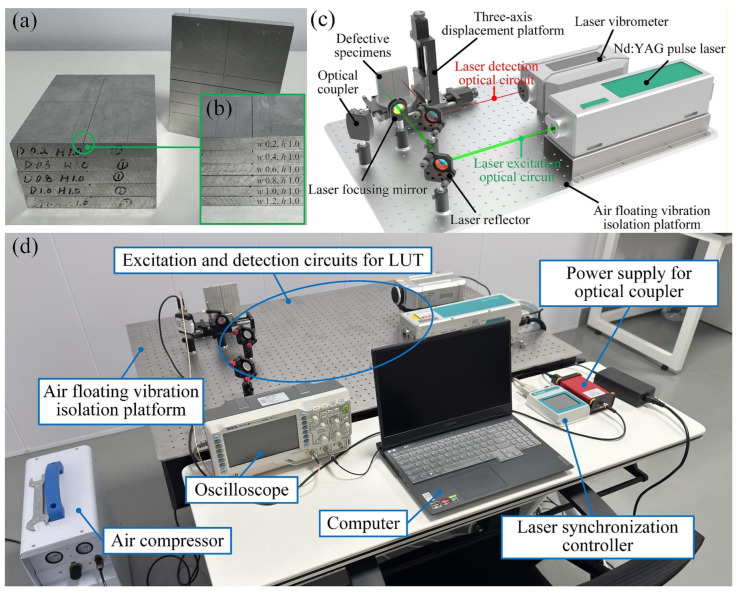
Specimens and laser-ultrasonic testing setup: (**a**) specimen set with labeled surface notches; (**b**) close-up of representative notch regions; (**c**) excitation–detection optical paths; and (**d**) integrated platform and acquisition hardware. Reproduced with permission from Xu et al., Mechanical Systems and Signal Processing; published by Elsevier, 2026 [106].

Laser-generated ultrasound can be paired with different receiving mechanisms, which should not be treated as one configuration. In a laser–EMAT arrangement, the laser pulse generates the surface wave and a nearby magnet–coil assembly detects the induced dynamic electromagnetic response. By contrast, the system shown in Figure 12 uses optical generation and laser-vibrometer reception: a pulsed Nd:YAG laser operating in the thermoelastic regime generates broadband surface waves, and an independent laser Doppler vibrometer records out-of-plane displacement [106]. The excitation and detection beam paths are arranged on an optical table with reflectors and focusing optics, while the specimen is positioned on a three-axis stage. A synchronization controller triggers the laser, vibrometer, and oscilloscope for coherent acquisition. The reported setup uses a 1064 nm Nd:YAG pulse (8 ns, 10 Hz) with 20 mJ pulse energy, and records the vibrometer output using an MSOX oscilloscope with 100 MHz bandwidth and 1 GSa/s sampling. These parameters describe the cited controlled laboratory system rather than a field-ready rail platform.

**Figure 13:**
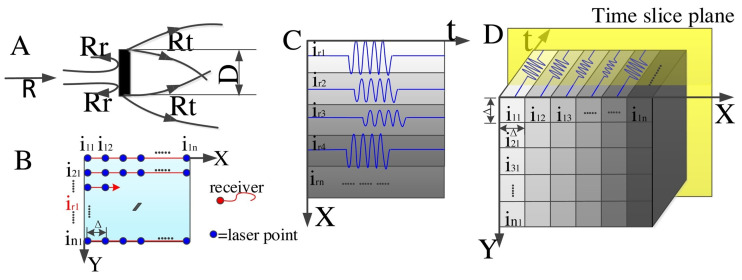
Surface-wave excitation and crack interaction under the laser thermoelastic effect: (**A**) Interaction of the surface wave R with a surface crack of length *D*; (**B**) Schematic of scanning-mode laser ultrasonic inspection; (**C**) Time-domain characteristics of scanning signals; (**D**) Wave-field slices F(x, y, t). Reproduced with permission from Jiang et al., Optik; published by Elsevier, 2021 [107].

Laser-ultrasonic inspection has limitations, including high equipment cost and sensitivity to optical alignment and surface condition. Jiang et al. proposed a non-contact quantitative method for oblique rail-head cracks using variational mode decomposition [107]. The method analyzes time-domain propagation images and surface-wave energy distributions to visualize rolling-contact-fatigue cracks of different lengths; the reported quantitative error was below 5% under the study conditions. Figure 13A defines a surface crack of length D and an incident Rayleigh wave R, which produces a reflected component Rr and a crack-interacting transmitted/diffracted component Rt. In Figure 13B, the pulsed laser scans an *n* × *n* grid at fixed spatial interval Δ and temporal interval ΔT while the receiving point remains fixed. Figure 13C represents the resulting three-dimensional data F(x, y, t). Time slices of F provide the spatial wave field, and sequential slices form a propagation animation used to identify the surface-connected crack. The reported error is source-specific and should not be interpreted as a general field sizing accuracy.

Laser ultrasonics combines optical excitation or reception with ultrasonic wave propagation, enabling non-contact, broadband, and spatially localized measurements [108]. Depending on the generated mode, receiving mechanism, and access geometry, it can be applied to the rail head, web, or base and can interrogate surface-connected, shallow subsurface, or deeper internal discontinuities [109,110,111]. This flexibility does not remove practical constraints: line of sight, optical reflectivity, vibration, stand-off, and surface contamination all affect signal quality. General reviews describe wider material and industrial applications [112,113], whereas the rail-specific evidence remains dominated by controlled specimens and prototype systems. The method is therefore treated here as a targeted characterization route rather than a geometry-independent, line-speed solution.

Pathak et al. detected rail foot cracks using laser-induced guided waves and simulated the effect of different frequencies and sensor locations on the detection results. This study helps to determine the equipment specifications and sensor locations for rail foot crack detection [114]. LUT can excite multiple ultrasonic wave modes in the measured object, with frequencies reaching the MHz range [115]. Nan et al. investigated a novel LUT-based detection system for identifying rail surface cracks as small as 0.5 mm. This system uses high-energy laser pulses to generate Rayleigh waves, while an EMAT probe receives the wave signals. To improve the signal-to-noise ratio, the EMAT probe coil was designed as a four-layer printed circuit board (PCB) with a butterfly configuration. The results demonstrated that the lift-off distance between the EMAT probe and the rail steel sample should be less than 3 mm [116]. Lian et al. proposed a finite element method (FEM) for laser-generated ultrasound to quantitatively detect V-shaped cracks on rail surfaces. This method can effectively identify crack dimensions, with a relative positioning error of 1.39% and most relative quantitative errors within 8% [117].

However, laser ultrasound technology still has some limitations, such as low photoacoustic energy conversion efficiency, weak ultrasonic signals, and high detection equipment costs [118]. Additionally, quantitative detection of rail defects in thermoelastic mode is challenging. This is because the laser-excited body wave has low energy, resulting in weak diffraction signals and a low signal-to-noise ratio for defect detection [119]. This requires a signal-processing technique to improve the ultrasound signal-to-noise ratio. Existing research on rail defect detection is predominantly qualitative. However, qualitative detection does not provide specific defect data and may fail to accurately assess defect severity, potentially overlooking severe issues. Compared to other excitation methods, the performance of laser ultrasonic transducers is more susceptible to constraints imposed by rail surface conditions and harsh on-site environments. Parameters such as the selected laser’s wavelength, pulse width, and energy, the excitation mechanism, and the rail’s inherent characteristics directly affect the generation and propagation of laser-generated ultrasound. Furthermore, factors that cause optical path fluctuations, such as on-site vibrations or air turbulence, introduce significant noise. The instability and nonlinearity of laser-induced ultrasonic signals, combined with the surface roughness of the rail, severely hinder the analysis and identification of defects, consequently leading to unclear imaging and inaccurate quantitative detection [107].

### 3.6. Summary

Table 6 presents different ultrasonic testing methods employing various excitation modes for detecting superficial defects in railways, providing a comparative analysis of four methods across multiple dimensions, including detection depth, resolution, surface adaptability, inspection speed, and their advantages and disadvantages. However, Table 6 shows that no excitation category is universally best. Contact piezoelectric UT/PAUT provides the strongest evidence for quantitative confirmation when coupling is acceptable. EMAT avoids couplant but trades this advantage for lift-off sensitivity, lower SNR, EMI susceptibility, and limited in-service evidence. Active air-coupled UT is constrained by the air–steel impedance mismatch and lacks a general field false-alarm benchmark, while laser UT is attractive for remote, controlled measurements but remains sensitive to surface optical properties and vibration. Table 7 converts these trade-offs into a practical selection matrix; its size and depth bands are engineering guidance, not acceptance limits.

## 4. Technology–Application Matching for Shallow Subsurface Rail Inspection

### 4.1. Scenario-Specific Technical Frameworks

Surface waves offer the advantages of single-point excitation and long-distance detection, propagating within a depth range of twice their wavelength from the workpiece surface while maintaining extensive propagation distances. They are commonly employed for the detection of metal surface cracks [132]. Rayleigh-wave displacement is concentrated near the free surface and decays with depth. The effective interrogation depth is not a fixed multiple of wavelength; it depends on wavelength, mode content, defect geometry, attenuation, sensor response, and the SNR required for a decision. Surface waves are therefore useful for surface-connected and shallow subsurface cracks when their wave field overlaps the target depth, while deeper indications require an explicit transition to bulk-wave or phased-array ultrasonic verification.

Rolling-contact-fatigue (RCF) cracks usually initiate at or very near the rail-head surface and propagate obliquely. A reported surface crack length near 15 mm should not be interpreted as an equivalent crack depth. An authoritative RCF review reports typical depths of approximately 2–4 mm, depths up to about 8 mm in heavy-haul conditions, and a 4–8 mm range during hydraulically assisted propagation [8,133]. Active air-coupled UT and laser surface-wave UT are therefore not assigned reliable standalone coverage of an 8–15 mm depth range. For targets around 8–10 mm, low-frequency piezoelectric or EMAT surface/guided waves may provide screening when wavelength and mode are appropriately selected, but the indication should be confirmed by bulk-wave UT or PAUT. Damage extending toward 10–15 mm is treated as a deeper-transition problem for which conventional bulk-wave UT/PAUT is the primary method; robust standalone surface-wave coverage under representative in-service conditions has not been demonstrated. Standardization challenges persist, as divergent NDT regulations across operators/governments hinder the adoption of unified inspection protocols, underscoring the need for harmonized testing standards to enhance rail safety [134].

### 4.2. Technical Challenges and Mitigation Strategies

For contact piezoelectric surface-wave inspection, irregular rail geometry, coupling variation, and structural noise require reference calibration and adaptive processing. Active air-coupled UT removes the liquid couplant but faces a severe air–steel impedance mismatch: more than 99.99% of incident acoustic energy is reflected under ideal normal-incidence estimates, leaving only a very small transmitted fraction. A receiver gain of approximately 100 dB compensates electronic amplitude; it is not a 100 dB denoising improvement because signal, acoustic background, and electronic noise may all be amplified. Mariani and Lanza di Scalea evaluated probability of detection against probability of false alarm through scenario-specific ROC analysis in numerical models [124]. Full-scale passive air-coupled field tests later examined speed, sensor location, raw and reconstructed SNR, and repeat-run redundancy at speeds up to 80 mph [9]. However, passive wheel-excited monitoring is not equivalent to active air-coupled surface-wave excitation. The reviewed literature does not establish a general false-alarm rate, standardized denoising gain, or broad natural-defect validation for active air-coupled rail inspection; its field suitability is therefore classified as conditional rather than established.

EMAT and laser UT likewise require evidence to be separated from deployment claims. The HBVD-EMAT increases broadband surface-wave amplitude and supports controlled multi-defect localization, but deep-crack sensitivity and multi-site in-service validation remain limited [135]. DiffUT increased mean accuracy from 78.0% to 93.3% and recall from 66.0% to 91.5% in the reported ultrasonic B-scan task [136], but such within-study gains do not by themselves demonstrate generalization to natural shallow subsurface rail defects. Synthetic augmentation can reproduce the generator’s bias, underrepresent rare morphologies and correlated field noise, or leak specimen-specific information if splitting occurs after augmentation. A defensible evaluation should split data by physical specimen or rail section before augmentation, keep an independent real-only external test set, report performance separately for real and synthetic data, examine calibration and POD/PFA across sites and operators, and vary lift-off, speed, temperature, roughness, and defect morphology. Until these controls are reported, synthetic-data results should be interpreted as model-development evidence rather than proof of field robustness.

### 4.3. Future Development Trends of Ultrasonic Testing for Rail Defects

**Figure 14:**
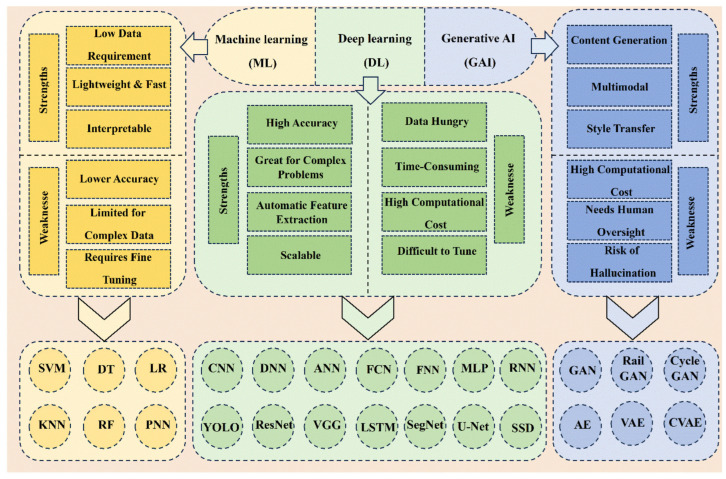
Strengths, weaknesses, and representative model families for machine learning, deep learning, and generative AI. Reproduced with permission from Khajehdezfuly et al., Journal of Industrial Information Integration; published by Elsevier, 2025 [140].

With the growing demand for intelligent railway maintenance, near-surface defect detection technologies in railways must continually make breakthroughs in sensitivity, adaptability, and cost-effectiveness to ensure the safe and efficient development of railway transportation. Future advancements will focus on intelligent systems, high precision, multi-technology integration, and deeper industrial applications. Future progress should be judged by validated inspection performance rather than by algorithmic speed alone. Multimodal workflows can combine rapid VT/MFL/ECT screening with targeted ultrasonic confirmation, while edge inference can reduce processing latency. In addition, data-driven analysis provides an effective approach for improving railway safety. Through the deep integration of algorithms such as dynamic graph neural networks and physics-informed neural networks, it is possible to extract ultrasonic echo signals and identify patterns [137,138]. However, millisecond-level classification does not remove the physical limits imposed by coupling, transduction efficiency, lift-off, wave-mode selection, and sensor coverage. Priority should therefore be given to natural-defect rail datasets, specimen- and site-wise data separation, calibrated uncertainty, common POD/PFA protocols, and independent multi-operator field trials. In the early decades, various machine learning (ML) algorithms, such as histogram matching, principal component analysis, support vector machines, and singular value decomposition, were employed to learn the characteristics of input data and perform analysis. The data used were diverse, including geometric measurements, vibrations, acoustic signals, and optical wave detection [139]. Figure 14 summarizes the broader strengths and weaknesses of ML, DL, and generative-AI models in railway monitoring.

In recent years, advancements in image processing technology, combined with the introduction of deep learning (DL) techniques and convolutional neural networks (CNNs), have led to the development of an increasing number of CNN-based methods for detecting surface defects. Machine learning models are lightweight, interpretable, and have low computational costs, typically used for statistical analysis, defect classification, and predicting railway track conditions by extracting features from sensor data. DL uses complex neural networks, such as CNNs, capable of identifying intricate patterns and automating the feature extraction process, and is highly effective in handling image, audio, and vibration data. In addition, CNNs can not only be used for image classification, but also for defect localization through bounding boxes. Such models can be efficiently deployed through transfer learning and pre-trained networks, reducing the demand for large amounts of training data and computational resources. However, the deployment of DL-based detection methods in real-world conditions faces practical challenges; for instance, studies have shown that environmental factors such as low illumination can reduce surface crack detection rates by approximately 50% compared to optimal conditions, underscoring the need for robust model design and data augmentation strategies [10]. GAI is a new generation of artificial intelligence that can generate synthetic data, augment training datasets, and create more advanced decision models, helping to improve the accuracy, flexibility, and innovativeness of monitoring systems [140].

## 5. Conclusions

Shallow subsurface rail-defect inspection requires sensing physics to be matched to defect depth and operational constraints; the reviewed evidence does not support a single universally superior method. Contact piezoelectric UT and PAUT provide the most mature basis for quantitative confirmation when coupling and surface preparation are acceptable. EMAT is attractive for non-contact low-frequency interrogation, but broader in-service validation, lift-off compensation, and standardized POD/PFA reporting remain necessary. Active air-coupled and laser-ultrasonic approaches show promise in controlled scenarios, yet the available evidence is insufficient to claim robust standalone coverage of natural defects throughout an 8–15 mm range at line speed. For the 8–10 mm transition, low-frequency surface/guided-wave screening should be confirmed with bulk-wave UT/PAUT; for 10–15 mm targets, bulk-wave UT/PAUT is the primary choice. Because reported sizes, speeds, SNR values, and resolution values were obtained under heterogeneous conditions, they are source-conditioned evidence rather than normalized rankings. Future work should use common rail specimens, natural-defect datasets, specimen- and site-wise data separation, calibrated uncertainty, and tiered screening–confirmation workflows with explicit probability-of-detection and false-alarm metrics.

## Figures and Tables

**Table 1:** Parameters Affecting MFL Performance.

Parameter Categories	Specific Parameters
testing equipment parameters	magnetization current
	probe lift-off value
defect characteristics	defect orientation
	defect depth and width
	defect location
component condition	material magnetic properties
	surface layer
operational and environmental conditions	scanning speed
	electromagnetic interference

**Table 2:** Parameters affecting MPI performance.

Parameter Categories	Specific Parameters
testing equipment parameters	magnetization current
	magnetization method (circumferential magnetization, longitudinal magnetization, multi-directional magnetization)
magnetic particle medium	type of magnetizing current
	particle type, suspension concentration
	defect characteristics
workpiece condition	material magnetic properties
	workpiece surface condition
operational and environmental conditions	viewing conditions

**Table 3:** Parameters affecting ECT performance.

Parameter Categories	Specific Parameters
testing equipment parameters	excitation frequency
	probe lift-off
	coil type
workpiece properties	electrical conductivity
	magnetic permeability
	workpiece geometry
defect characteristics	defect depth, location, orientation, size

**Table 4:** Parameters affecting UT performance.

Parameter Categories	Specific Parameters
acoustic and equipment parameters	frequency
	amplitude
	probe angle and type
	inspection sensitivity
defect characteristics	defect orientation
	defect size and shape
	defect nature
workpiece condition	material acoustic properties
	workpiece geometry
inspection conditions and coupling	coupling condition

**Table 5:** Source-conditioned evidence for NDT methods relevant to shallow subsurface rail-defect workflows.

Method	Role in a Shallow-Subsurface Workflow	Source-Specific Reported Operating Evidence	Defect and Test Context	Key Characteristics
MPI [28,32,34]	stationary confirmation of surface-breaking and very shallow indications	sensitivity decreases rapidly with burial depth.	manual or automated bench inspection; ferromagnetic material; prepared surface and magnetic particles.	Intuitive display; low cost; operator-dependent, polluting
ECT [45,52,53,72]	screening of surface-connected and shallow subsurface discontinuities	a vehicle system operated at 5–30 km/h with lower speed recommended to reduce vibration [52];	different probes, artificial anomalies, lift-off, rail condition, and decision thresholds.	Non-contact; portable; skin effect, lift-off sensitive
Conventional UT [64,73,74,75]	quantitative confirmation and deeper-defect assessment	Under laboratory conditions, the conventional UT detection speed is 40–80 km/h, with real-world speeds potentially of 15 km/h [64,74].	predominantly internal-defect and controlled rail tests;	Well-established; requires couplant
MFL [23,72,76,77]	rapid screening of magnetic indications from surface-connected or shallow subsurface damage	Jia et al. proposed MFL system at 60 km/h on actual track damages [23]; The MFL testing system that is mounted on the GTC-80X rail flaw detection vehicle can reach up to 180 km/h [72];	ferromagnetic rail; laboratory, numerical, and vehicle studies use different magnetization, lift-off, and defect geometries.	No couplant; high sensitivity; requires magnetic saturation
VT [71,78,79,80]	high-speed screening of visible crack mouths, shelling, spalling, and wear	image-based systems for large surface-visible defects have been reported at up to 400 km/h [71]; The China Academy of Railway Sciences proposed an onboard track detection system operate at speeds of up to 160 km/h [79];	vehicle-mounted cameras; performance depends on illumination, contamination, motion blur, and visible defect expression.	Highly efficient; low cost; requires AI training data

**Table 6:** Merits and limitations of surface-wave excitation techniques for ultrasonic NDT of shallow subsurface rail defects.

Excitation Method	Piezoelectric Transducer [120,121,122]	Air-Coupled [123,124,125,126]	Electromagnetic [127,128,129]	Laser Ultrasonic [130,131]
**Detection depth**	0.5–10 mm	1~5 mm	0.5~10 mm	4~8 mm
**Resolution**	0.1~0.8 mm	0.1~1.2 mm	0.1~0.73 mm	0.1~1 mm
**Coupling requirements**	Requires couplant	No couplant	No couplant	No couplant
**Detection speed**	40~120 km/h	40–130 km/h	<15 km/h	<40 km/h
**Frequency range**	0–500 kHz	0.75–2 MHz	0.5~10 MHz	5–100 Mhz
**Excitation efficiency**	High	Low	moderate	Low
**SNR**	Highest, Direct contact provides stable acoustic coupling with minimal noise interference	Low, >99.9% wave energy reflected at air-solid interface; echo signals require amplification	Moderate, due to low energy conversion efficiency; 3 mm lift-off required to achieve sufficient SNR	Low. Low photoacoustic conversion efficiency yields weak signals and low SNR; signal processing required
**Lift off**	Must make contact	Relying on the air path causes increased signal amplitude attenuation	Increasing the separation leads to a significant reduction in signal amplitude (≤3 mm in practical applications)	Relatively speaking, it can operate at larger intervals, but is limited by optical reflectivity.
**Field rail inspection applicability**	Surface cleaning is mandatory before inspection, with sensitivity to oil contamination.	Exhibits superior tolerance to oil contamination and corrosion layers	Robust tolerance to oil/corrosion, susceptible to EMI degradation	Requires rigorous surface cleaning, critically dependent on optical reflectivity

Notes: Values are source-conditioned examples and are not normalized performance limits. Resolution and minimum detectable size depend on wavelength, defect geometry, threshold, and SNR. NR denotes not reported. Receiver amplification is not equivalent to denoising gain. Active air-coupled excitation and passive air-coupled reception are treated as different configurations.

**Table 7:** Evidence-graded decision matrix for shallow subsurface rail-defect inspection.

Target Depth and Indicative Defect Scale	Inspection Task/Speed	Surface/Coupling Condition	Dominant Noise	Recommended Method	Evidence and Confirmation
surface-visible to 2 mm; crack mouth or small surface-connected feature	network screening; low to high acquisition speed	variable contamination and roughness	lighting, motion, lift-off	VT + ECT or MFL screening	use ultrasonic confirmation for crack-front depth; VT alone cannot establish subsurface extent.
0.5–2 mm; sub-mm to about 2 mm	targeted inspection, generally ≤15 km/h or stationary sizing	clean surface; couplant acceptable	low to moderate structural noise	contact piezoelectric Rayleigh-wave UT or PAUT	best-supported quantitative route; confirm threshold using a rail-specific reference block.
1–5 mm; small to medium (about 0.5–3 mm)	Screening at moderate speed followed by low-speed sizing	Couplant unacceptable or oily surface	EMI and lift-off variation	EMAT for targeted inspection; active air-coupled UT only in controlled studies	confirm safety-critical indications with contact UT/PAUT. Air-coupled field FAR/POD remains unestablished.
4–8 mm; usually ≥1 mm characteristic depth	low-speed targeted characterization	Clean optical path or stable small lift-off	vibration, roughness, optical-path or EMI noise	low-frequency piezoelectric UT/PAUT or EMAT; laser UT under controlled conditions	evidence is method- and specimen-specific; use bulk-wave confirmation when defect orientation is uncertain.
8–10 mm;	targeted low-speed verification	variable field surface	high structural and operational noise	low-frequency piezoelectric or EMAT guided-wave screening plus PAUT/bulk-wave UT	surface-wave-only evidence is limited. A screen–confirm workflow is required.
10–15 mm transition band; downward-turning RCF or incipient transverse defect	targeted safety-critical verification	any; surface preparation as needed	mode conversion and structural echoes	conventional bulk-wave UT or PAUT as the primary method	outside the best-supported shallow surface-wave range; air-coupled and laser surface-wave UT are not reliable standalone choices.

Note: Defect-size bands are indicative characteristic dimensions derived from heterogeneous studies; they are not regulatory acceptance thresholds. Method qualification should use rail-specific reference defects and report probability of detection and probability of false alarm.

## Data Availability

No new data were created or analyzed in this study. Data sharing is not applicable to this article.

## Appendix A. Review Positioning, Search Strategy, and Evidence-Selection Record

The contribution of this review is a bounded critical synthesis rather than a claim of new sensing physics. Xiong et al. [3] organize rail inspection broadly into ultrasonic, electromagnetic, and visual methods and propose an integrated detection guideline, but internal, surface-visible, and subsurface defects are considered together and source-specific performance values are not converted into a depth-stratified selection rule. Ge et al. [64] emphasize guided-wave modes, propagation, and long-range rail monitoring rather than comparing four surface-wave excitation routes for the first few millimeters beneath the running surface. General laser-ultrasonic reviews [112,113] address wider material-characterization and industrial applications, while railway-AI reviews [10,139] focus on data-driven maintenance across infrastructure and do not compare transduction physics. The present review therefore adds value by (i) defining the shallow-subsurface scope and the 10–15 mm hand-off boundary, (ii) separating surface-screening evidence from subsurface-characterization evidence, (iii) tracing numerical claims to their source conditions instead of treating heterogeneous values as normalized, and (iv) providing a decision matrix that links depth, defect scale, speed, surface condition, noise, and confirmation need. A structured comparison is provided in Table A1.

**Table A1:** Critical positioning of the present review against recent adjacent reviews.

Review	Primary Scope	Main Synthesis	Gap Relative to the Present Review
Xiong et al. [3]	broad rail-defect inspection	ultrasonic, electromagnetic, and visual methods; integrated guideline	does not isolate shallow subsurface defects or convert source-conditioned values into a depth-stratified decision matrix.
Ge et al. [64]	rail guided waves	modes, propagation, transducers, and long-range monitoring	does not critically compare contact piezoelectric, active air-coupled, EMAT, and laser surface-wave excitation for the shallow zone.
Lian et al. and Xie et al. [112,113]	laser ultrasonics across materials and industries	generation, reception imaging and industrial applications	rail shallow-subsurface validation, operational constraints, and cross-method selection are not the organizing focus.
Sridharan et al. and Khajehdezfuly et al. [10,139]	ai-enabled railway monitoring	data-driven inspection and maintenance across railway assets	does not compare ultrasonic transduction physics or define evidence-based depth hand-offs.
Present review	shallow subsurface rail defects	source-conditioned evidence for screening and characterization, four ultrasonic excitation routes, and deployment matching	adds an explicit scope definition, an 8–15 mm hand-off discussion, evidence limitations, and a practical decision matrix.

For reproducibility, the two complete English strings are designated Q1 and Q2 solely to avoid repeating the same literal text in every database wrapper. Q1 = (Rail or Railway or Rail track) and (Shallow subsurface defect or Near-surface defect or Surface-connected crack or Rolling contact fatigue or Shelling or spalling) and (Nondestructive test or Non-destructive evaluation or Ultrasonic or Surface wave or Rayleigh wave or EMAT or Electromagnetic acoustic transducer or Air-coupled ultrasonic or Laser ultrasonic or Magnetic flux leakage or Magnetic particle inspection or Eddy current or Visual inspection). Q2 = (Rail or railway) and (Ultrasonic or Surface wave or Rail defect) and (Deep learning or Machine learning or Automatic defect recognition or Data augmentation).

The field wrappers applied to the complete strings above were: Web of Science Core Collection Advanced Search, TS = (Q1) and TS = (Q2); Scopus Advanced Search, TITLE-ABS-KEY (Q1) and TITLE-ABS-KEY (Q2); IEEE Xplore, Q1 and Q2 in All Metadata; and ScienceDirect, Q1 and Q2 in Title, Abstract, or Author-Specified Keywords. The exact targeted Google Scholar queries were: “rail” “shallow subsurface defect” ultrasonic; “rail” “rolling contact fatigue” “surface wave”; “rail” EMAT “surface crack”; “rail” “air-coupled ultrasonic”; “rail” “laser ultrasonic” crack; and “rail defect” ultrasonic “deep learning”. The first 200 relevance-ranked results of each targeted query were screened. The exact CNKI query is reproduced in Figure A1. All searches used the 2000–2026 date interval and the final update date recorded for this revision was 13 July 2026.

Backward citation searching was applied to key rail-NDT and guided-wave reviews. Google Scholar and backward searching were supplementary discovery routes rather than controlled bibliographic databases. Screening records were consolidated by title, DOI, and first author/year. Core screening selected 137 references; the final update added three targeted supplementary sources [8,9,10], producing the 140-item bibliography. The combined pre-full-text exclusion count is reported because a defensible database-specific duplicate subtotal was not retained; this limitation prevents a claim of full PRISMA compliance.
